# Femoral vessel complications after transfemoral TAVR—A contemporary sonography‐based assessment of 480 patients with third‐generation transcatheter valves

**DOI:** 10.1002/clc.24197

**Published:** 2023-12-09

**Authors:** Paul Werner, Martin Winter, Markus Müller, Bernhard Zierfuss, luliana Coti, Markus Mach, Sabine Scherzer, Paul Simon, Günther Laufer, Andrea Willfort‐Ehringer, Martin Andreas

**Affiliations:** ^1^ Department of Cardiac Surgery Medical University of Vienna Vienna Austria; ^2^ Division of Angiology, Department of Internal Medicine II Medical University of Vienna Vienna Austria

**Keywords:** femoral artery stenosis, postinterventional sonography, transfemoral aortic valve replacement, vascular complications

## Abstract

**Background:**

Postinterventional sonographic assessment of the femoral artery after transfemoral transcatheter aortic valve replacement (TF‐TAVR) has the potential to identify several pathologies. We investigated the incidence and risk factors of femoral vessel complications in a modern TAVR collective using postinterventional sonography.

**Methods:**

Between September 2017 and March 2022, 480 patients underwent TF‐TAVR with postinterventional femoral sonography at a single center. Clinical outcomes and adverse events were analyzed after the Valve Academic Research Consortium 3 (VARC‐3) criteria.

**Results:**

In this cohort (51.2% male; age 80 ± 7.5 years, median EuroSCORE II 3.7) 74.8% (*n* = 359) were implanted with a self‐expandable and 25.2% (*n* = 121) with a balloon‐expandable valve. The main access (valve‐delivery) was located right in 91.4% (*n* = 438), and the primary closure system was Proglide in 95% (*n* = 456). Vascular complications (VC) were observed in 29.16% (*n* = 140) of patients; 23.3% (*n* = 112) presented with minor‐ and 5.8% (*n* = 28) with major VC. Postinterventional femoral artery stenosis on the main access was observed in 9.8% (*n* = 47). Multivariable logistic regression analysis revealed female sex (*p* = .03, odds ratio [OR] 2.32, 95% confidence interval [CI] 1.09–4.89) and the number of used endovascular closure devices (*p* = .014, OR 0.11, 95%CI 0.02–0.64) as predictive factors for femoral artery stenosis.

**Conclusions:**

The incidence of postinterventional femoral artery stenosis following TF‐TAVR was higher than expected with a number of used closure devices and female sex being independent risk factors. Considering the continuous advance of TAVR in low‐risk patients with preserved physical activity, emphasis should be directed at the correct diagnosis and follow‐up of these complications.

## Graphical abstract

Key Question:

What is the incidence of vascular complications (VC) after TF‐TAVR (*n* = 480) when using a sonography‐based postinterventional assessment?

The incidence of VCs remains high, and femoral artery stenosis was more frequently detected than expected.

Take‐Home Message:

Postinterventional femoral vessel sonography should be performed routinely after TF‐TAVR as it has the potential for detection of relevant access‐related complications

TF‐TAVR, transfemoral aortic valve replacement; VC, vascular complication.

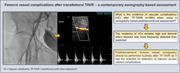

## 1 INTRODUCTION

Transcatheter aortic valve replacement (TAVR) has become the leading therapeutic intervention for the treatment of aortic stenosis in the elderly and is recommended as first‐line treatment in patients above the age of 75 years.^1^ Recent studies indicated a survival benefit of the transfemoral (TF) approach compared to alternative approaches.^2^, ^3^ Increased patient comfort, shorter hospital times, and procedural ease consolidated the transfemoral route as the preferred and recommend access.^1^

Although TF‐TAVR offers a favorable risk profile, vascular access complications remain a matter of concern and are associated with increased morbidity.^4^, ^5^ Several patient‐related risk factors have been identified for vascular complications (VC), including female sex, pre‐existing peripheral vascular disease, and iliofemoral vessel tortuosity and calcification.^6^, ^7^, ^8^, ^9^, ^10^ Procedural‐related risk also played a role in VC as well as operator experience.^6^, ^7^ Earlier cohorts reported up to 15% of patients suffering from major VC,^4^ whereas numbers of major VC ^11^, ^12^ improved in later cohorts. The decrease of VC rates is most likely attributable to the downsizing of the commonly used access sheaths. Nevertheless, inconsistent definitions of VC and different postinterventional diagnostics remain problematic. Current guidelines make no recommendations on postinterventional assessment of the access site following TF‐TAVR.^1^, ^13^

As TF‐TAVR continues to evolve rapidly and more low‐risk patients are being treated with transcatheter procedures, incidence and timely diagnosis of VC become more important in this collective. For this reason, we aimed to investigate the incidence of VC in a contemporary real‐world TAVR collective and underlying risk factors of such. A comprehensive approach integrating peri interventional angiographic and postinterventional sonographic imaging of the femoral vessels was therefore applied.

## 2 PATIENTS AND METHODS

### 2.1 Patients and Study‐Design

Between September 2017 and March 2022, 592 patients underwent TAVR in a surgically lead TAVR program. Patients were enrolled in The Victory II registry, which is an ongoing prospective registry at the Medical University of Vienna including all patients who undergo TAVR at our institution. Patients with other access than transfemoral and without postinterventional sonography were excluded from this analysis. Postinterventional sonography was performed on Day 1 after the procedure. All Duplex examinations were performed by experienced sonographers at a specialized Duplex laboratory (Division of Angiology, Department of Medicine 2). in transverse and longitudinal views and included the common‐, profound, and superficial‐ femoral artery. In the case of proximal femoral artery complications, a sonography of the iliac arteries was performed. A Resona 7 System (Mindray) with a 7 MHz linear array transducer, was used. Angle‐corrected spectral Doppler waveforms were acquired from longitudinal images. Diagnostic criteria for hemodynamically significant stenosis were used according to current guidelines.^14^

Study endpoints were defined according to the Valve Academic Research Consortium 3 (VARC 3) for aortic valve interventions.^15^ The primary endpoint was vascular complication, stratified into major and minor, depending on the patient outcome resulting in death, severe bleeding, amputation, ischemia, or irreversible end‐organ damage or not. Bleeding was stratified into Type 1–4, depending on the need for intervention, amount of red blood cell transfusions, hemoglobin drop, end‐organ failure, and death as a result. Postinterventional stenosis was defined as a relevant flow acceleration in the target vessel exceeding 3 m/s (corresponding to stenosis exceeding 50% of vessel diameter) or delta peak systolic velocity > 1.4 m/s (difference between the peak systolic velocity [psv] at the stenotic site and the prestenotic segment).^16^, ^17^

### 2.2 Interventional technique

Transfemoral TAVR was performed under moderate sedation and local anesthesia. Arterial and venous femoral access for pigtail and temporary pacemaker placement (“off‐access”) was established without imaging guidance for puncture. The main access puncture was performed under fluoroscopic guidance using a digital subtraction angiography overlay image with live fluoroscopy. Two endovascular closure devices (Perclose ProGlide™, Abbott Laboratories) were deployed. Following valve implantation, the main access was closed, and angiography was performed to detect bleeding, pseudoaneurysm, stenosis, or dissection. The off‐access was closed using a collagen‐sponge‐based closure 6‐French closure device (Angio‐Seal™ VIP).

### 2.3 Statistical methods

Continuous variables were described by mean ( ± standard deviation) or median [quartiles] in case of non‐normal distributions. Continuous normal distributed data are presented as mean and standard deviation and median and first and third quartiles for non‐normal distribution.

Univariate risk analysis for predictors of VC and femoral artery stenosis was performed using a logistic regression model. Statistically significant predictors and known risk factors were further used for the creation of a multivariable logistic regression model. For inclusion in the multivariable model, the α‐level was set at 0.1. No correction for multiple testing was performed. Two‐sided *p*‐values < .05 were considered statistically significant. Statistical analysis was performed using IBM SPSS Statistics 27 (V. 27, released 2019; IBM Corp.) and MATLAB (R2015b, MathWorks).

## 3 RESULTS

Of 592 patients who underwent TAVR between September 2017 and March 2022, 480 patients were included in the final analysis. Sixty‐eight patients were excluded due to an alternative access route, and 44 patients were excluded due to insufficient or missing documentation of postinterventional sonography of the femoral vessels. The mean age in the group was 80 ± 7.5 years, 51.2% (*n* = 246) were males and the median EuroSCORE II was 3.7% (1.8%; 5.9%). Preoperative characteristics are summarized in Table 1.

**Table 1:** Preinterventional patient characteristics in the overall study cohort.

Variables	Overall Cohort
	*n* = 480
Age (years)	79 ± 7.5
Sex (male)	246 (51.2%)
BMI (kg/m²)	27.2 ± 5.1
EuroSCORE II (%)	3.7 [1.8; 5.9]
Arterial hypertension	432 (90%)
Diabetes	147 (30.6%)
Dyslipidemia	389 (81.6%)
Peripheral artery disease	53 (11%)
Chronic lung disease	86 (17.9%)
History of smoking	126 (26.3%)
Dialysis	12 (2.5%)

Abbreviation: BMI, body mass index.

All patients underwent transfemoral TAVR as described in the methods section. Self‐expandable valves (SEV) were implanted in 74.8% (*n* = 359), and balloon‐expandable valves (BEV) were implanted in 25.2% (*n* = 121). The main access (sheath for valve deployment) was located on the right side in 91.6% (*n* = 440) of cases. The endovascular suture‐based closure device ProGlide was used as primary closure device in 95% (*n* = 456) of cases, and its newer iteration, the Prostyle (Abbott Laboratories) in 1.9% (*n* = 9). Other closure devices used as primary closure were the collagen and anchor‐based Manta device (Teleflex) in 2.5% (*n* = 12) and the InSeal device (InSeal Medical) in 0.2% (*n* = 1) of procedures. The number of endovascular closure devices primarily used was two in 94.6% (*n* = 452) of cases, one in 2.7% (*n* = 13), three in 1.9% (*n* = 9), and four in 0.4% (*n* = 2).

The primary closure device used for the off‐access (pigtail insertion via 6‐French sheath) was the 6 French Angio‐Seal™ (Terumo Interventional Systems) in 82.9% (*n* = 398) of procedures. Other devices used were the Femoseal device (Terumo Interventional Systems) in 10.2% (*n* = 49), the 8 French Angio Seal device, (Terumo Interventional Systems) in 4% (*n* = 19) of procedures, the Prostyle device in 0.4% (*n* = 2) and no closure in 0.4% (*n* = 2). Procedural details are summarized in Table 2.

**Table 2:** Procedural details of the overall study cohort.

Variables	Overall cohort
	*n* = 480
Valve type	
BEV	121 (25.2%)
SEV	359 (74.8%)
Valve model	
Sapien S3/S3 Ultra	43/78 (9%/16.3%)
Evolut/Pro/Pro Plus	103/52/24 (21.5%/10.8%/5%)
Portico/Navitor	122/40 (25.4%/8.3%)
Accurate/Neo	4/6 (0.8%/1.3%)
Allegra	4 (0.8%)
Jena Valve	4 (0.8%)
Valve sizes BEV	121 (25.2%)
23	33 (27.3%/121)
26	50 (41.3%/121)
29	38 (31.4%/121)
Valve sizes SEV	359 (74.8%)
23	29 (8.1%/359)
25	39 (10.9%/359)
26	25 (7%/359)
27	68 (18.9%/359)
29	105 (29.2%/359)
31	1 (0.3%/359)
34	78 (21.7%/359)
Main access (right groin)	438 (91.3%)
Primary closure device type	131 [107;164]
ProGlide/Prostyle	457/9 (95.2%/1.9%)
Manta	12 (2.5%)
InSeal	1 (0.2%)

Abbreviations: BEV, balloon‐expandable valve; SEV, self‐expandable valve.

### 3.1 Vascular complications and interventions

In the overall cohort, 140 (29.16%) of patients suffered from VC. Of those, 112 (23.3% of overall cohort) were minor and 28 (5.8% of overall cohort) were major VC. The greater part (*n* = 17, 3.5%) of major VC were vascular injuries leading to VARC 3 bleeding ≥ 2, while the rest of cases (*n* = 11; 2.3%) were device closure failures leading to VARC 3 bleeding ≥ 2. Bleeding complications were observed in 218 patients (45.4%), and 88 patients (18.3%) presented with bleedings greater than type‐1; 81(16.9%) patients suffered from type‐2 bleedings and seven patients (1.5%) suffered from type‐3 bleedings. No type four bleedings were observed in the study cohort.

**Figure 1:**
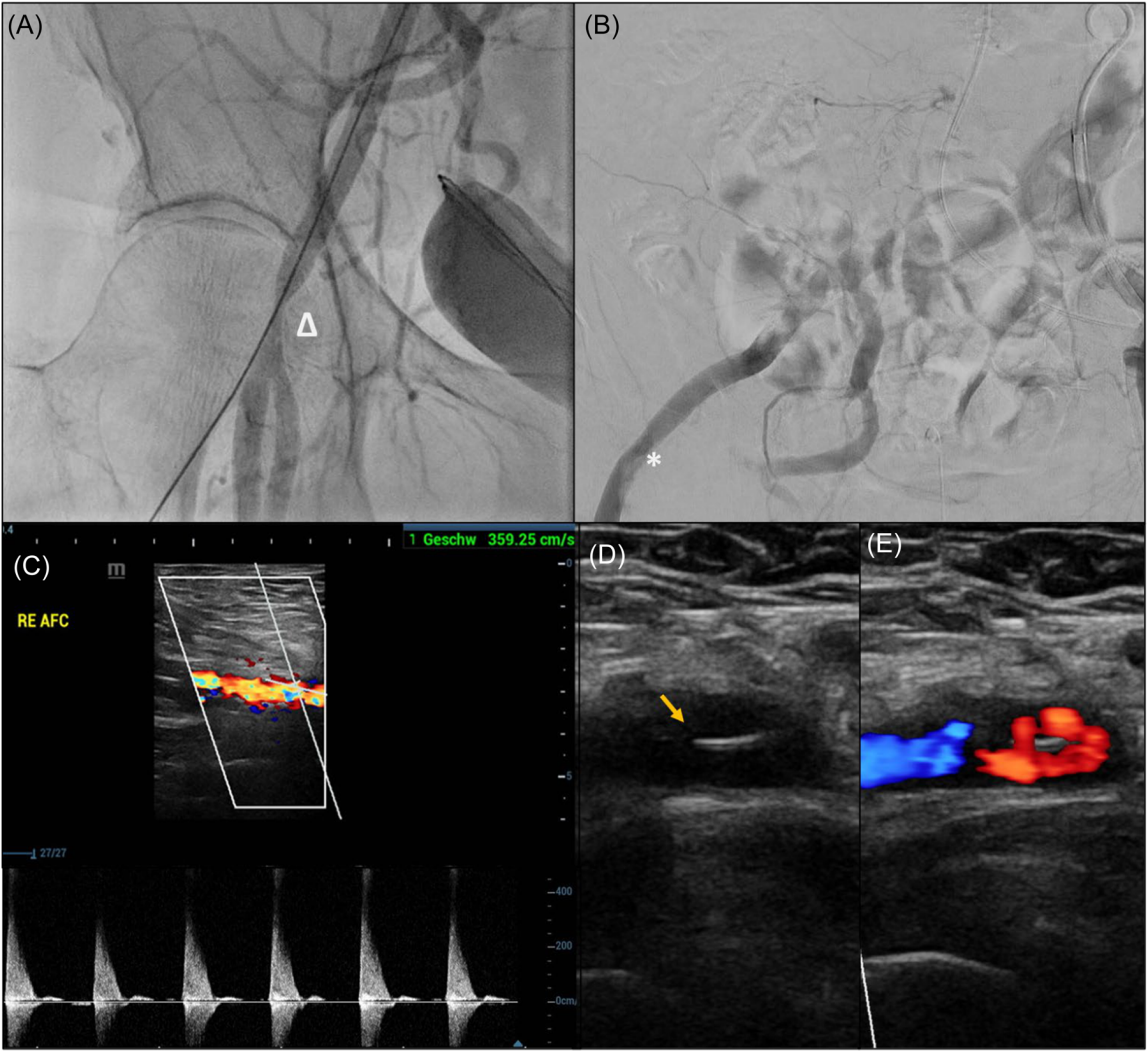
Imaging of femoral arteries after transcatheter aortic valve replacement (TAVR) with stenotic flow profile (A) fluoroscopy of femoral artery stenosis after endovascular closure (Δ) (B) fluoroscopic view of a femoral artery dissection after endovascular closure (*) (C) sonography of a stenotic flow profile of the femoral artery (D) sonography of dissection membrane (arrow) (E) color flow profile of femoral artery dissection.

Postinterventional femoral artery stenosis at the main access was observed in 47 patients (9.8%) (Figure 1,A,C). In this subgroup, the mean peak flow velocity at the main access common femoral artery was 4.47 ± 1.04 m/s and the mean delta peak systolic velocity was 3.31 ± 1.17 m/s. Patients who presented with femoral artery stenosis following TAVR underwent immediate intervention (same session) in six cases and late intervention in one case, whereas the rest of patients (*n* = 40) underwent conservative management with regular follow‐ups in the outpatient clinic. Postinterventional stenosis at the off‐access was observed in 27 patients (5.6%) with a mean peak systolic velocity of 4.01 ± 1.04 m/s and a delta peak systolic velocity of 3.61 ± 3.06 m/s.

Pseudoaneurysms were observed in 11 patients (2.3%) at the main access. A larger proportion of patients (*n* = 19, 4%) presented with a postinterventional pseudoaneurysm at the off‐access. Iatrogenic arteriovenous fistula was reported in four cases (0.8%) at the main access and in seven cases (1.5%) at the off access. Femoral artery dissection was observed in 19 cases (4%) at the main access and in four cases (0.8%) at the off access (Figure 1,B,D,E). Postinterventional hematoma was observed in 44 patients (9.2%) at the main access and in 16 patients (3.3%) at the off access.

**Figure 2:**
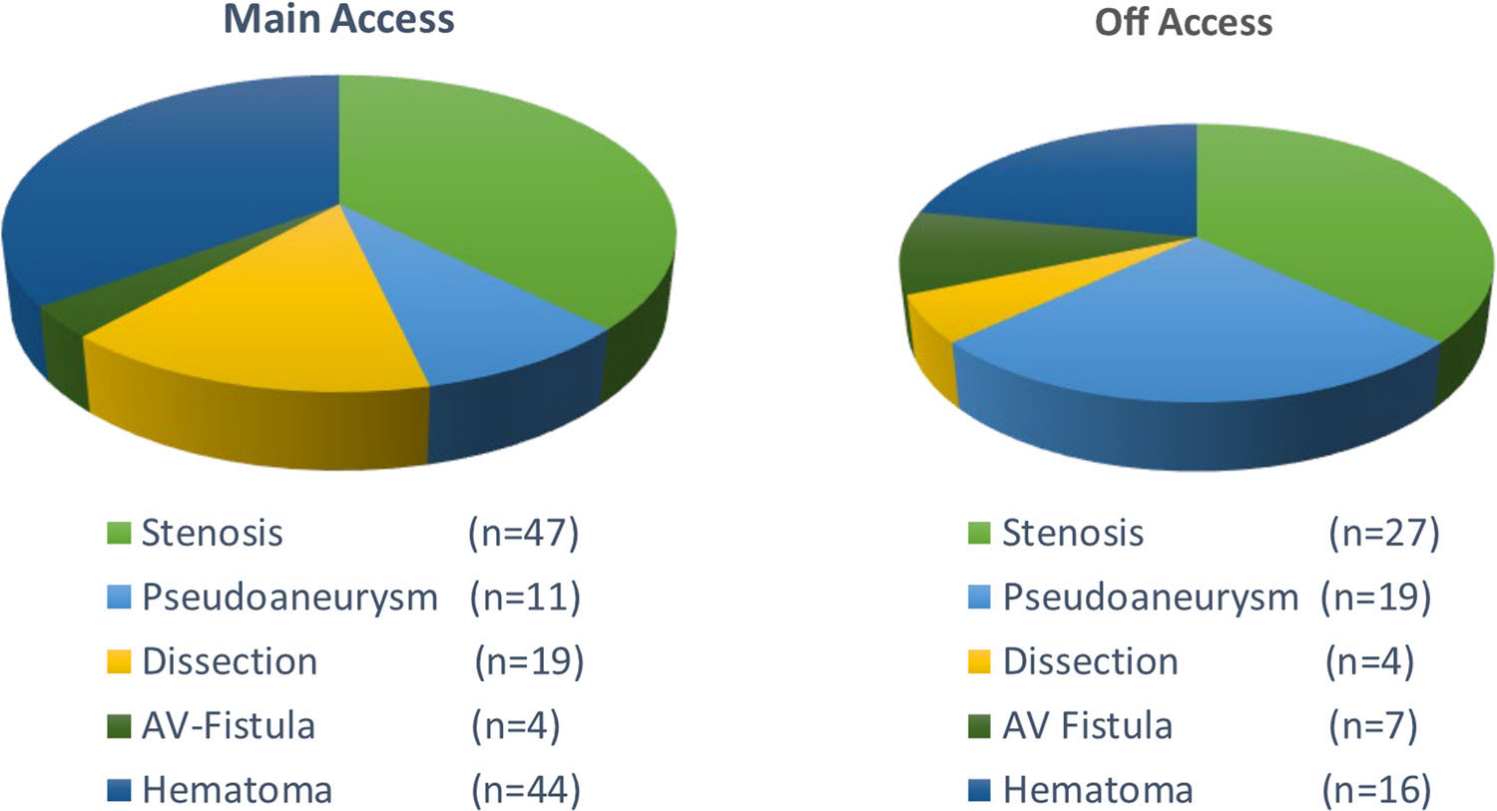
Femoral artery complications stratified after main access and off access site.

Intervention of the iliac and femoral vessels after TAVR was required in 30 patients (6.3%), with 16 patients (3.3%) requiring surgery and 14 patients (2.9%) undergoing endovascular procedures. The indication for the intervention of the iliac‐femoral vessels was the presence of a pseudoaneurysm in 12 patients (2.5%), iatrogenic stenosis in eight cases (1.7%) uncontrollable bleeding in nine cases (1.9%), and femoral artery dissection in one case (0.2%). Femoral vessel complications are summarized in Table 3 (Figure 2).

**Table 3:** Postoperative morbidity of the overall study cohort.

Clinical events	Overall cohort
	*n* = 480
Vascular complications	131 (29.1%)
Minor	112 (23.3%)
Major	28 (5.8%)
Bleeding events	
Type 1	130 (27.1%)
Type 2	81 (16.9%)
Type 3	7 (1.5%)
Type 4	0
Main access	
Stenosis	47 (9.8%)
Pseudoaneurysm	11% (2.3%)
Dissection	19 (4%)
Atrio‐venous fistula	4 (0.8%)
Hematoma	44 (9.2%)
Off access	
Stenosis	27 (5.6%)
Pseudoaneurysm	19 (4%)
Dissection	4 (0.8%)
Atrio‐venous fistula	7 (1.5%)
Hematoma^a^	16 (3.3%)

### 3.2 Risk factors

Several patient characteristics, such as comorbidities and procedural data were used for univariate logistic regression analysis of VC. History of smoking (*p* = .013, OR 1.74, 95% CI 1.13–2.69) was identified as the only risk factor. A multivariable model was created, including history of smoking, peripheral artery disease, sex, prosthesis size, valve model, and side of used access. In this model, smoking history was still a significant predictor of VC (*p* = .007, OR 1.85, 1.19–2.93).

For postinterventional femoral artery stenosis at the main access, univariate logistic regression analysis identified female sex (*p* < .001, OR 3.05, 95% CI 1.56–5.93), number of used closure devices (*p* = .009; OR 0.22, 95% CI 0.07–0.68) and size of the used prosthesis (*p* = .002, OR 0.84, 95% CI 0.76–0.94) as predictive factors. A multivariable logistic regression including sex, number and type of used closure devices, size of used prosthesis and peripheral vascular disease identified female sex (*p* = .03, OR 2.32, 95% CI 1.09–4.89) and the number of used endovascular closure devices (*p* = .014, OR 0.11, 95% CI 0.02–0.64) as predictive factors for femoral artery stenosis (Table 4).

**Table 4:** Univariable and multivariable logistic regression models to identify risk factors for femoral artery stenosis.

Prognostic factor	Univariable models		Multivariable model	
	OR [95% CI]	*p* value	OR [95% CI]	*p* value
Sex (female)	3.05 [1.57–5.94]	.001	2.32 [1.1–4.9]	.028
Peripheral vascular disease	2.08 [0.94–4.58]	.07	2.05 [0.09–4.65]	.086
Prosthesis size (mm)	0.002 [0.76–0.94]	.002	0.9 [0.8–1.02]	.086
Type of closure device	0.91 [0.52–1.60]	.75	0.56 [1.45–2.19]	.41
Nr. of closure devices	0.22 [0.07–0.68]	.009	0.12 [0.02–0.65]	.014

Abbreviations: CI, confidence interval; OR, odds ratio.

## 4 DISCUSSION

This study investigated the incidence and risk factors in a contemporary transfemoral TAVR cohort. and presents with the following principal findings: (1) The incidence of VC remains considerable in a real‐world TAVR cohort with third‐generation devices. (2) Femoral artery stenosis was prevalent in a larger than expected proportion of patients after TF‐TAVR and female sex and number of used closure devices were identified as independent risk factors. (3) Postinterventional femoral artery sonography is a useful and feasible modality to identify access‐related complications in TF‐TAVR.

Vascular complications were frequently reported in the earlier TAVR cohorts with first or second‐generation TAVR devices. In a prospective cohort of 127 patients undergoing TF‐TAVR with the Sapien valve (Edwards Lifesciences), VC were observed in 35 patients (27.6%), with 22 cases (17.3%) of major VC and 13 cases (10.2%) of minor VC (defined after VARC 1 criteria).^6^ An incidence of 2.4% for femoral vessel stenosis/occlusion, 4.7% for femoral vessel dissection, and 1.6% for pseudoaneurysms was reported. No differentiation regarding observed side (main access/off access) was reported.^6^ In a propensity‐matched multicentric study (CONTROL) comparing endovascular closure devices (ProGlide vs. Prostar XL), 944 patients were analyzed. Vascular complications ranged from 19.9% (ProGlide) to 22.2% (Prostar XL) between groups, with major VC accounting for 1.9% (ProGlide) and 7.4% (Prostar XL), respectively.^5^ Femoral artery stenosis was significantly more prevalent in the Proglide group with 3.4% versus 0.6% in the Prostar group. Data from the German Aortic Valve Registry (GARY) showed a time‐dependent reduction of severe VC over the years with the current incidence of those remaining at approximately 4%.^18^ A more recent single‐center cohort of 878 patients who underwent TAVR with third generation devices reported 17.3% VC (VARC‐2) with 9.9% of major VC with femoral stenosis or occlusion observed in 3.3% and dissection and pseudoaneurysms observed in 2.2% and 2.3% cases, respectively.^19^

In this study, a relevant number of VC was observed, with 139 cases (29%). Importantly, in contrast to the GARY registry and other trials, all patients were routinely screened for VC via sonography after the index procedure in addition to intraoperative angiographic control. Major VCs were observed in 5.8%, which is notably lower than first or second‐generation TAVR cohorts ^4^, ^6^ and also compared to more recent ones.^19^ An important role in the continuous decline of major VC rates is attributable to the reduction of sheath diameters and to the addition of advanced periprocedural imaging techniques such as fluoroscopic overlay.

Femoral artery stenosis was observed in nearly 10% of cases at the main access (*n* = 47, 9.8%) and in 5.6% at the off access in this study. This is almost threefold as high as in other collectives described in the literature, where it ranges from 0.5% to 3%.^5^, ^19^ The increase in incidence was attributed to the postinterventional sonographic examination, as it allows for exact evaluation of the interventional site. Femoral vessel stenosis after transvascular interventions with larger sheaths is probably an underdiagnosed entity. In patients presenting with peri‐interventional femoral artery stenosis, same session intervention was performed in 12.8%, whereas late intervention was performed only in 2.1%, and conservative management was followed in 85.1%. The large number of patients with conservative treatment (or no follow‐up with the vascular specialist) might be explained by the older age of this cohort and the asymptomatic stenosis. As TAVR is continuously pushing into a younger patient collective, where one could assume a higher level of activity, active monitoring, and early femoral intervention might become more relevant.

Another underreported pathology following femoral access is the formation of pseudoaneurysms, which we observed in 2.3% (*n* = 11) at the main access and with a higher incidence of 4% (*n* = 19) at the off access. Patient‐ and procedure‐related factors affect this outcome; (1) the anatomically lesser attractive side is often chosen as the off access; (2) puncture at the off access was performed under no imaging guidance; (3) in contrast to the main access, the off access is not controlled angiographically after vascular closure; and (4) endovascular closure at the off access was mostly performed with a collagen sponge based closure device. A reduction in this complication might offer a greater patient benefit, as formation of pseudoaneurysms is often associated with surgical reintervention.

To our knowledge, this is one of the first reports of systematic postinterventional assessment utilizing femoral artery duplex sonography in a modern transfemoral TAVR cohort. Therefore, it is more likely to report the true incidence of certain complications compared to other studies not applying the described approach. Nevertheless, this data set is a single‐center study and therefore represents a specific subset of surgeons over a specific time period.

## 5 CONCLUSION

A comprehensive, sonography‐based postinterventional assessment algorithm in patients who underwent transfemoral TAVR presents a useful method to correctly assess the incidence of vascular complications. The incidence of postinterventional femoral artery stenosis following TF‐TAVR was higher than expected with a number of used closure devices and female sex being independent risk factors. Considering the continuous advance of transcatheter valve implantation in low‐risk patients with preserved physical activity, emphasis should be directed at the correct diagnosis and follow‐up of these complications.

## CONFLICT OF INTEREST STATEMENT

Martin Andreas is proctor/speaker/consultant (Abbott, Edwards, Medtronic, Boston, Zoll) and received institutional funding (Abbott, Edwards, Medtronic, LSI). Guenther Laufer is an advisory‐board member (Edwards). The other authors have no conflict of interest to declare.

## Data Availability

The data underlying this article will be shared on reasonable request to the corresponding author.
